# Atraumatic Orbital Emphysema in a Young Woman

**DOI:** 10.5811/cpcem.1897

**Published:** 2024-03-26

**Authors:** Eladio Albornoz, Janet Wildemuth, Josephine Valenzuela

**Affiliations:** * Arizona State University, Department of Medical Studies, Phoenix, Arizona; † Valleywise Health, Departments of Emergency Medicine and Internal Medicine, Phoenix, Arizona; ‡ Creighton University of Phoenix, Phoenix, Arizona; § University of Arizona Phoenix, Phoenix, Arizona

**Keywords:** *atraumatic orbital emphysema*, *point-of-care ultrasound*, *case report*

## Abstract

**Case Presentation:**

We describe the presentation, evaluation, and management of a young female patient presenting to the emergency department with atraumatic orbital emphysema, a rare condition. This patient was diagnosed using point-of-care ultrasound and computed tomography and was managed expectantly.

**Discussion:**

Atraumatic orbital emphysema is a rare clinical condition more common in early middle-aged female patients with certain historical features such as chronic sinusitis, facial surgery or trauma, tobacco smoking, or current upper respiratory symptoms. While most cases will resolve spontaneously, rarely this condition can lead to vision-threatening orbital compartment syndrome, requiring lateral canthotomy or needle decompression.

Population Health Research Capsule What do we already know about this clinical entity?

*Orbital emphysema is an uncommon clinical condition usually caused by trauma, though atraumatic etiologies have been reported as well.*

What is the major impact of the images?

*While examples of orbital emphysema are common, this is the first published example of a point-of-care ultrasound of this condition.*

How might this improve emergency medicine practice?

*Familiarity with this condition may prompt the clinician to select suitable imaging, screen for orbital compartment syndrome, and consult a specialist.*

## CASE PRESENTATION

**Image 1.**
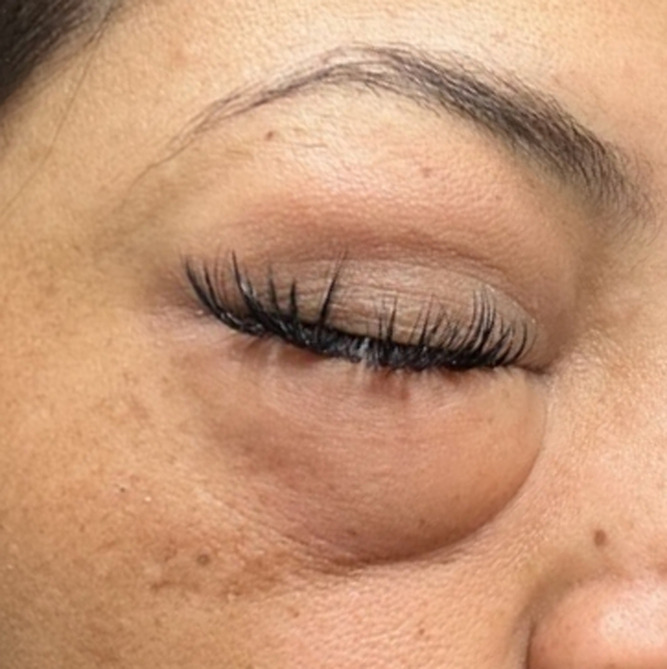
Periorbital swelling of the right eye in a female patient.

A 36-year-old woman presented to the emergency department with pain and swelling around her right eye after blowing her nose the evening prior (Image 1). She denied headache, fever, eye discharge, and visual changes. She denied any history of trauma, recent surgery, or upper respiratory symptoms. Physical examination was notable for periorbital swelling. There was crepitus to palpation of the area. Pupils were equal, round, and reactive to light. Visual acuity was 20/30 in both eyes, with an elevated intraocular pressure in the affected eye of 32 millimeters of mercury (mm Hg) (reference range 10–20 mm Hg), but without proptosis. Eye movements were normal and painless. There was no conjunctival injection, hemorrhage, tearing, or discharge from the eye.

**Image 2.**
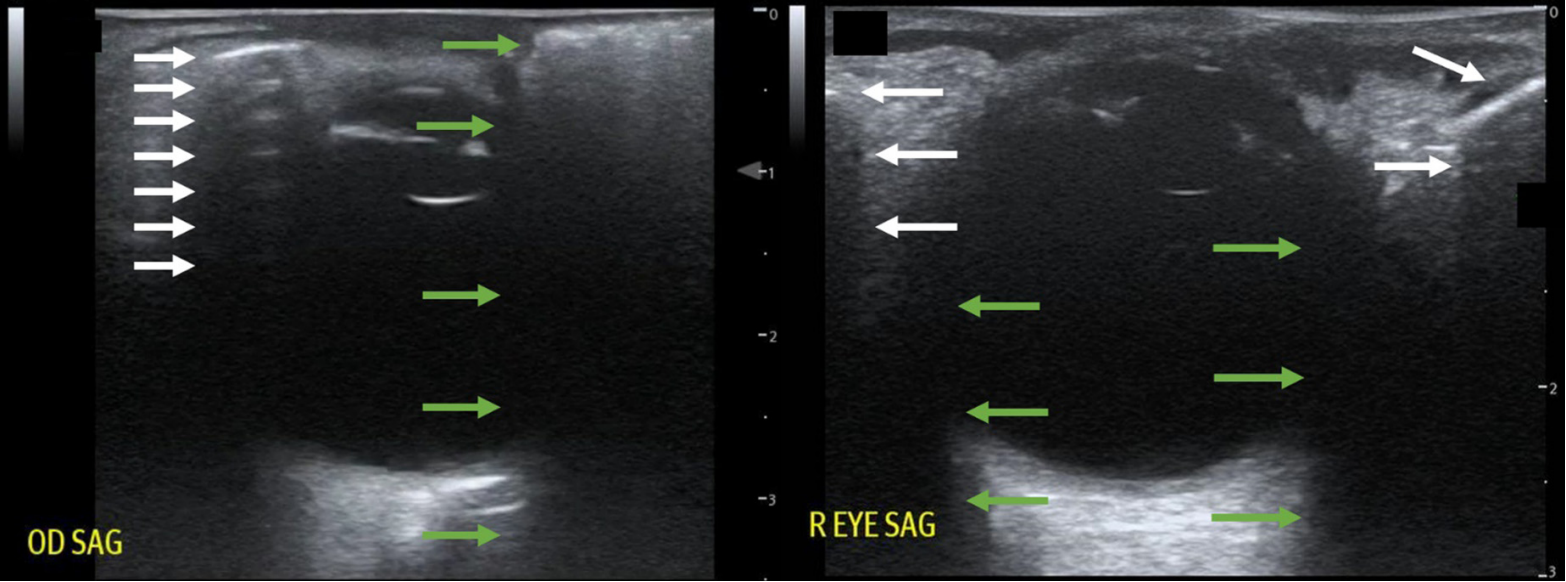
Point-of-care ultrasound of the patient’s eye (left) compared with a normal eye ultrasound (right). In the patient’s ultrasound on the left, a hyperechoic line is seen just below the skin surface (top white arrow), which represents the air-tissue interface of subcutaneous emphysema. Below are equally spaced, repeating lines of reverberation artifact (additional white lines), typical of highly reflective air-tissue interfaces, analogous to “A-lines” on normal lung ultrasound. Comparatively, in the right image, the shadow of the bony orbit in a normal eye ultrasound (white arrows) originates deeper below the skin surface, is sharply demarcated at the edges, and lacks reverberation artifact. Also shown in the patient’s ultrasound on the left is the “dirty shadowing” associated with subcutaneous air (green arrows.). Compared to the shadows of edge artifact indicated by the green arrows in the normal ultrasound on the right, these “dirty shadows” originate from an irregular surface, appear more “smeared,” and are not well-demarcated at the edges.

**Image 3.**
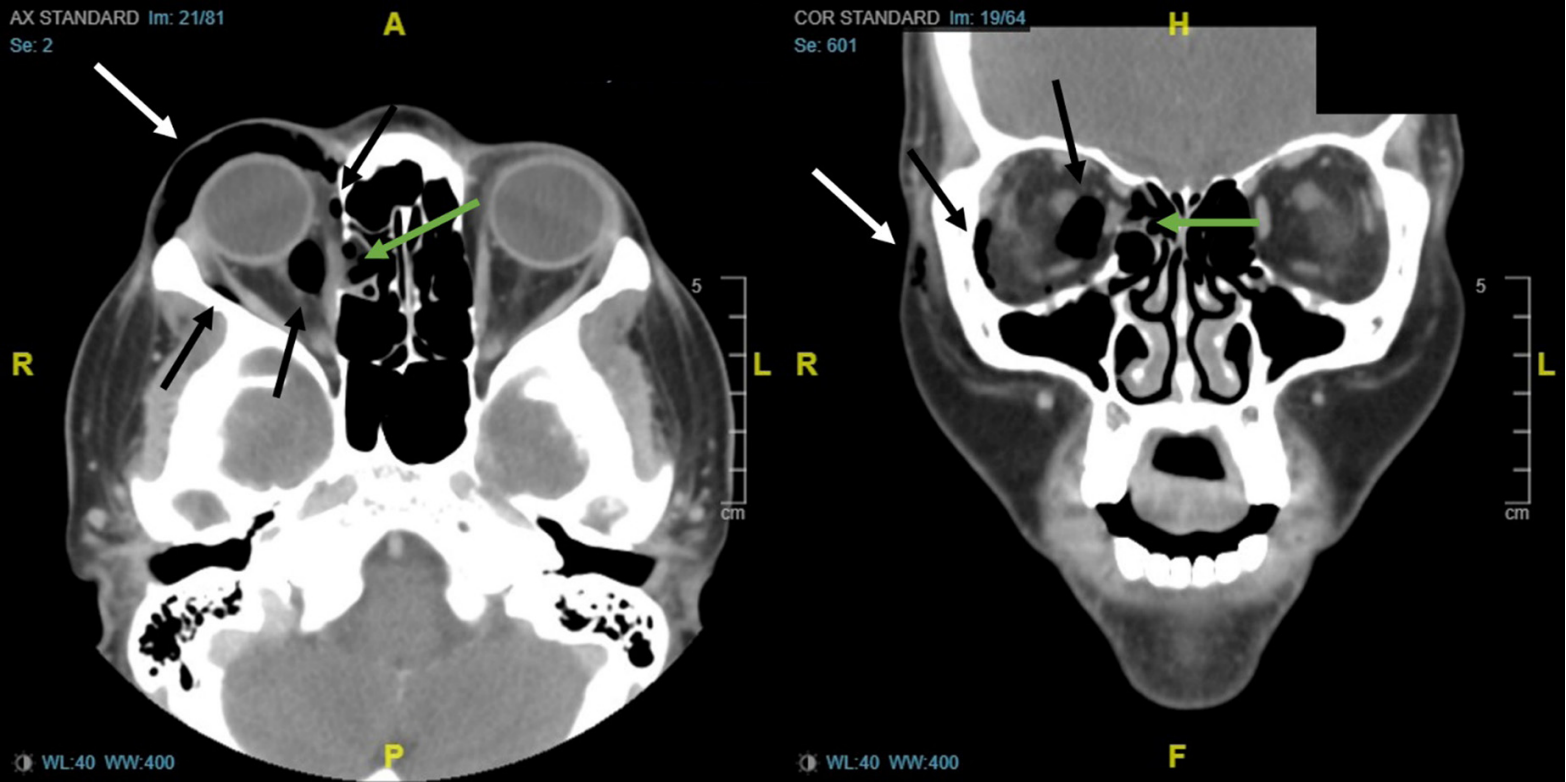
Computed tomography of the facial bones, axial view on the left and coronal view on the right, demonstrating subcutaneous periorbital emphysema (white arrows), intraconal orbital space emphysema (black arrows), with a defect in the lamina papyracea of the ethmoid sinus (green arrows).

The patient was laid supine, and a point-of-care ultrasound was performed with a linear transducer (Image 2). Notable findings included “dirty shadowing” and ring-down artifact consistent with air in the periorbital soft tissue. Computed tomography (CT) confirmed a defect in the lamina papyracea of the ethmoid sinus (the medial orbital wall), with air trapped in the orbit (Image 3).

## DISCUSSION

Orbital emphysema is an uncommon condition that results from trapping of air in the orbit and periorbital tissue.^1^ Typical signs and symptoms include periorbital swelling, crepitus, pain, proptosis, chemosis, vision changes, and relative afferent pupillary defect.^1^ It is normally associated with trauma, although dozens of other etiologies have been reported, including sneezing, nose-blowing, coughing, postoperative complication, and use of a continuous positive airway pressure device.^1^^–^^3^ Non-traumatic etiologies are more common in patients who are early middle-aged, female, and have a history of facial trauma, surgery, sinusitis, or tobacco smoking, or have current upper respiratory symptoms.^4^ It is theorized that chronic inflammation and/or remote trauma can weaken the lamina papyracea of the ethmoid sinus, so that positive pressure then causes a fracture and air entry into the orbit.^2^ The differential diagnosis of atraumatic orbital emphysema should include orbital cellulitis, malignancy, orbital foreign body, and hematoma.^1^ Apart from age and gender, this patient had no additional risk factors.

When suspected on history and physical examination, a diagnosis is made on CT of the face. Point-of-care ultrasound findings demonstrating air in the subcutaneous tissue can increase the index of suspicion for this entity but cannot definitively establish the diagnosis. Regarding management, most cases resolve in 7–10 days spontaneously. In rare cases, orbital compartment syndrome can develop, with trapped air compressing either the optic nerve itself or the ophthalmic artery, causing ischemia. This is a vision-threatening complication requiring emergent lateral canthotomy and cantholysis or needle decompression.^5^ Orbital compartment syndrome is detected by increased intraocular pressure and abnormal visual acuity on physical examination.^5^

In this case, the patient’s intraocular pressure was only mildly elevated, with normal visual acuity; thus, our on-call ophthalmologist and plastic surgeon recommended expectant management. The patient followed up in plastic surgery clinic three days later with significantly reduced pain and swelling. She was asked to continue to follow sinus precautions, including no nose-blowing, sneezing with mouth closed, straw usage, diving, flying on airplanes, or smoking, for several more weeks.
